# Impact of the COVID-19 pandemic on treatment for mental health needs: a perspective on service use patterns and expenditures from commercial medical claims data

**DOI:** 10.1186/s12913-023-09080-9

**Published:** 2023-02-16

**Authors:** Ta-Hsin Li, Leah Kamin, Judy George, Fernando Suarez Saiz, Pablo Meyer

**Affiliations:** 1 IBM Thomas J. Watson Research Center, Yorktown Heights, NY 10598-0218 USA; 2 IBM Watson Health, Cambridge, MA 02142 USA

**Keywords:** COVID-19, Depression, Eating disorders, Healthcare cost, Services utilization, Substance use

## Abstract

**Objective:**

To examine changes in use patterns, cost of healthcare services before and after the outbreak of the COVID-19 pandemic, and their impacts on expenditures for patients receiving treatment for depression, anxiety, eating disorders, and substance use.

**Methods:**

This cross-sectional study employed statistical tests to analyze claims in MarketScan® Commercial Database in March 2020-February 2021 and quarterly from March 2020 to August 2021, compared to respective pre-pandemic periods. The analysis is based on medical episodes created by the Merative™ Medical Episode Grouper (MEG). MEG is a methodology that groups medical and prescription drug claims to create clinically relevant episodes of care.

**Results:**

Comparing year-over-year changes, proportion of patients receiving anxiety treatment among all individuals obtaining healthcare services grew 13.7% in the first year of the pandemic (3/2020–2/2021) versus 10.0% in the year before the pandemic (3/2019–2/2020). This, along with a higher growth in price per episode (5.5% versus 4.3%) resulted in a greater increase in per claimant expenditure ($0.61 versus $0.41 per month). In the same periods, proportion of patients receiving treatment for depression grew 3.7% versus 6.9%, but per claimant expenditure grew by same amount due to an increase in price per episode (4.8%). Proportion of patients receiving treatment for anorexia started to increase 21.1% or more in the fall of 2020. Patient proportion of alcohol use in age group 18–34 decreased 17.9% during the pandemic but price per episode increased 26.3%. Patient proportion of opioid use increased 11.5% in March–May 2020 but decreased or had no significant changes in subsequent periods.

**Conclusions:**

We investigated the changes in use patterns and expenditures of mental health patients before and after the outbreak of the COVID-19 pandemic using claims data in MarketScan®. We found that the changes and their financial impacts vary across mental health conditions, age groups, and periods of the pandemic. Some changes are unexpected from previously reported prevalence increases among the general population and could underlie unmet treatment needs. Therefore, mental health providers should anticipate the use pattern changes in services with similar COVID-19 pandemic disruptions and payers should anticipate cost increases due, in part, to increased price and/or service use.

**Supplementary Information:**

The online version contains supplementary material available at 10.1186/s12913-023-09080-9.

## Introduction

The impact of COVID-19 pandemic and related public health, social and economic measures on mental health conditions have attracted attention from public health researchers and the media [1–19]. Much of the current research describes the epidemiological impacts of COVID-19 on mental health without describing the actual medical system use patterns and their financial implications during the pandemic [1]. This study adds to that literature by identifying changes in use patterns and expenditures for patients receiving services for selected common mental health conditions. These conditions are depression, generalized anxiety disorder, anorexia and bulimia eating disorders, alcohol and opioid use disorders. These mental health conditions are chosen because of public interest and their sufficiently large sample size.

The COVID-19 pandemic has broadly impacted health service utilization patterns in commercially-insured populations [20]. Notably, there has been significant reductions in preventive healthcare and elective surgeries [20, 21]. These service impacts may be the result of patient fears of infection and reduced or disrupted access to health services [19]. Unlike preventive health and elective surgeries, there is evidence that mental health services use increased during the pandemic as a proportion of total service use [7–10].

This cross-sectional analysis uses anonymized patient-level medical claims data from MarketScan® Commercial Database. This data set is one of the largest commercial claims data sets in the US. It is contributed by commercial insurers and covers a large population of employees and their dependents who are geographically dispersed across the U.S [22]. The analysis of this data set adds insight and clarity to the impact of the COVID-19 pandemic on mental health services use during the pandemic time periods compared to pre-pandemic time periods.

The findings presented here are potentially useful for health insurers to anticipate mental health service changes driven by system-wide disruptions like the COVID-19 pandemic. The findings are also potentially useful for service providers involved with reforming mental health service delivery to address the lasting impacts of the COVID-19 pandemic. Additionally, researchers can use these findings to support hypothesis generating research.

## Methods 

The methods described below were originally designed for continuous surveillance and monitoring of changes in all medical conditions and services based on claims data [20]. All analyses were performed by a proprietary Python-based software developed to support IBM Watson Health’s clients.

### Sampling and grouping

The study design considers 12-month time windows covering or containing a period of interest. For each time window, we analyzed a random sample of 5 million individuals enrolled in a non-capitated healthcare plan for at least one month within the time window; the study included all claims incurred and paid within the time window.

Medical and pharmacy claims within each time window were grouped into medical episodes using the Merative™ Medical Episode Grouper (MEG). MEG is a grouping methodology based on primary diagnosis codes together with a consideration of time proximity and clinical relevance [23, 24] (see supplementary material).

By grouping claims into mental health episodes, we detect changes in the proportions of patients receiving mental health services among all individuals with a claim (claimants), the prices per episode, and the episodes per patient, by comparing them in various time periods during the pandemic with the respective periods before the pandemic. Impacts of these changes on the per claimant expenditure are also calculated.

Motivated by public interests, especially regarding mental health conditions of young people during the pandemic due to restrictions such school closing and social distancing, we further break down the population by commonly-used age groups.

### Nomenclature and key performance indices

For each target or reference period, we focus on enrollees with at least one claim in this period for any reason; we call them *claimants*. The expenditure on a mental health condition, denoted as *Cost,* is defined as the total allowed amount in the claims for treating patients with this condition, normalized by the number of claimants; it represents the contribution of this condition to the total per claimant expenditure on all conditions.

We consider three factors in use patterns and expenditures that potentially drive the changes of Cost in a time period: (1) price per episode, or *Price*, defined as the allowed amount per episode of the condition, (2) episode intensity, or *EPR* (episode-to-patient ratio), defined as the number of episodes per patient, and (3) patient proportion, or *PCR* (patient-to-claimant ratio), defined as the proportion of patients being treated for the condition among all claimants.

For each factor, the *change* (or trend) of this factor in a target period relative to a reference period is measured by the difference of observed values in these periods as a fraction (or percentage) of the observed value in the reference period, i.e., change in target period = (value in target period – value in reference period) ÷ value in reference period. The *financial impact* of this change, which measures the contribution of this change to the increase or decrease in Cost, is defined as the expected Cost when this factor takes on the observed value in the target period while the remaining factors are held at their values in the reference period, minus the observed Cost in the reference period (see supplementary material for details).

For each factor, the *excess change* of this factor in a pandemic period against a pre-pandemic period is defined as the change in the pandemic period (relative to its reference period) minus the change in the pre-pandemic period (relative to its reference period). Similarly, the *excess financial impact of change* in a pandemic period against a pre-pandemic period is defined the financial impact of change in the pandemic period (relative to its reference period) minus the financial impact of change in the pre-pandemic period (relative to its reference period). In this study, we use the excess change and excess financial impact of change to investigate the effect of the pandemic against a pre-pandemic baseline.

The financial impacts of these factors are additive components of the overall financial impact defined simply as the difference between Cost in the target period and Cost in the reference period. The residual of the overall financial impact after removing these components is attributable to interactions among the contributing factors. All financial impacts will be presented in the unit of US dollars per month as will be for Cost.

To detect changes in these factors, we employ a *Z-*test procedure for log ratios in the target period relative to the reference period [25–28] (see supplementary material).

### Target and reference periods

This study involves two types of analysis, yearly and quarterly. The yearly analysis provides a general view of trend. The quarterly analysis provides additional intra-year dynamics associated with different waves of the pandemic.

In the yearly analysis, we compare the changes in the 12-month pandemic period 3/2020–2/2021 with the changes in the pre-pandemic period 3/2019–2/2020. The changes in the pandemic period are calculated against the reference period 3/2019–2/2020 (i.e., one year prior to the pandemic period); the changes in the pre-pandemic period are calculated against the reference period 3/2018–2/2019 (i.e., one year prior to the pre-pandemic period). See Tables 2, 3, 4, 5, 6 and 7 for definitions of these periods.

In the quarterly analysis, we compare the changes in each of seven consecutive 3-month periods during the pandemic starting in 3/2020 until 11/2021 with the changes in a respective period before the outbreak of the pandemic. This respective pre-pandemic period is one year prior to the pandemic period if the latter is in the first year of the pandemic or two years prior to the pandemic period if it is in the second year of the pandemic. The changes in a pandemic period are measured against a pre-pandemic reference period one year or two years prior; the changes in a pre-pandemic period are measured against a reference period which is one year prior. Exact definitions of these periods can be found in Tables 8, 9, 10, 11, 12 and 13.

## Results

**Fig. 1:**
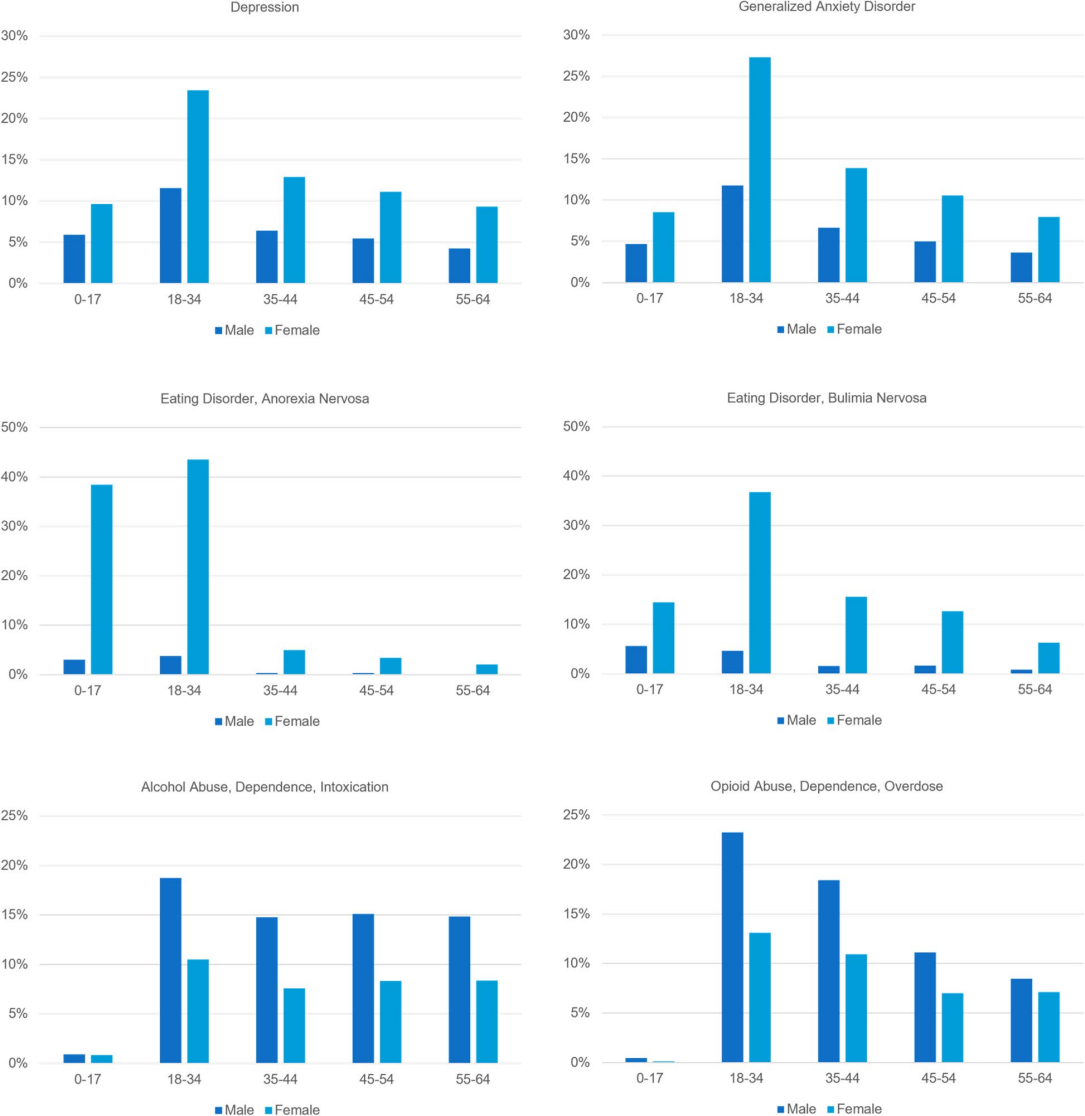
Distribution of patients by age group and gender

Table 1 contains the per claimant expenditure (Cost) of each mental health condition in the 12-month period 3/2020–2/2021 together with its contributing factors: price per episode (Price), episode intensity (EPR), and patient proportion (PCR). These quantities are obtained for patients in five age groups as well as for patients in all age groups combined. These general-purpose age groups are supplied by MarketScan®. Some age groups do not appear in Table 1 and the subsequent analysis because of small sample sizes or small expenditure amounts. Figure 1 shows the compete distribution of patients by age group and gender for each mental health condition. In this period, the per claimant expenditure for all physical and mental health conditions was $460.18 per month. Of this total expenditure, $8.12 was spent on depression, $3.63 on anxiety, $0.46 on anorexia, $0.19 on bulimia, $3.11 on treating patients for alcohol use, and $0.96 on treating patients for opioid use.

**Table 1:**
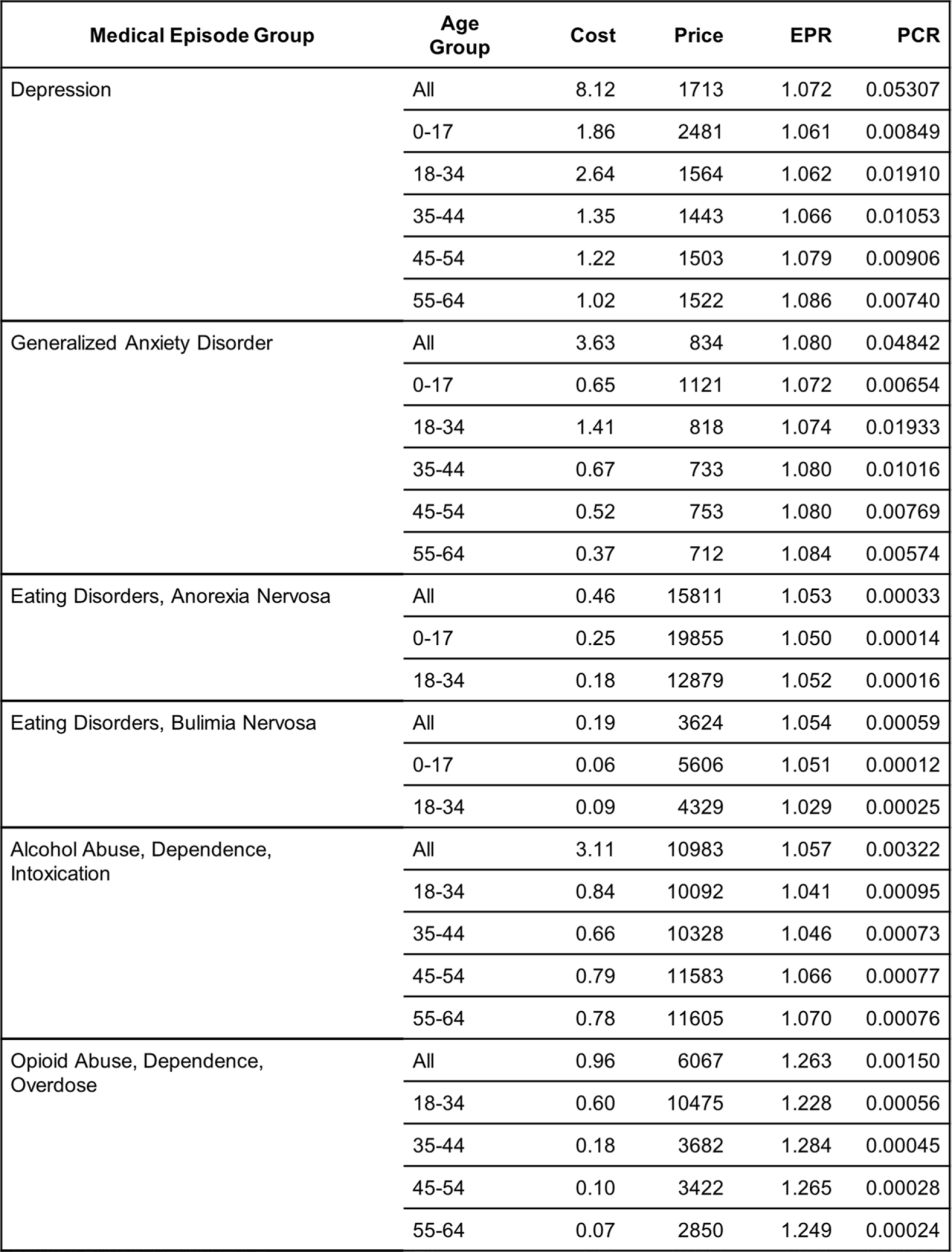
Cost and Its Contributing Factors of the Mental Health Episodes in the 3/2020–2/2021 Period

*Cos*t allowed amount per claimant (per month), *Price* allowed amount per episode, *EPR* number of episodes per patient, *PCR* proportion of patients among claimants

Tables 2, 3, 4, 5, 6 and 7 show the change (with 95% confidence interval) and the financial impact of change in the contributing factors together with the overall financial impact (Overall) for the six mental health conditions from the yearly analysis, where changes in 3/2020–2/2021 relative to 3/2019–2/2020 are compared with changes in 3/2019–2/2020 relative to 3/2018–2/2019.

**Table 2:**
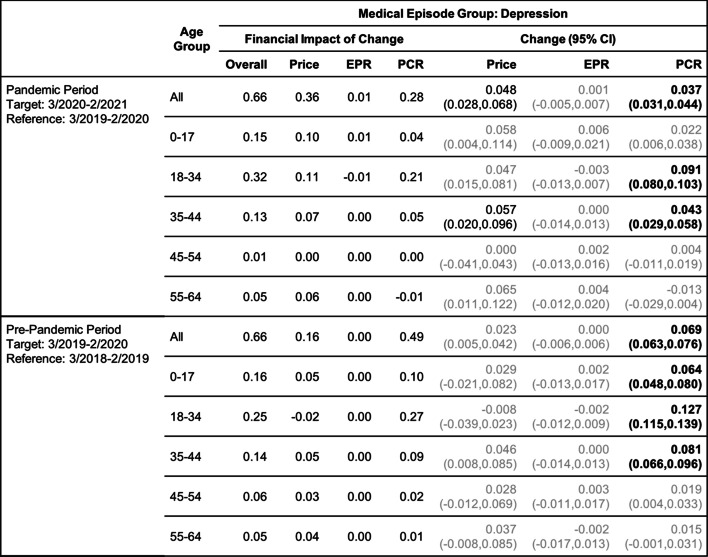
Changes of Mental Health Episodes in Twelve-Month Periods: Depression

Change in Price, EPR, and PCR: boldface, strong statistical significance (absolute value of Z score greater than 5, or *p*-value < 5.7e-7); lightface, weak or no statistical significance (absolute value of Z score less than or equal to 3, or *p*-value > 0.0027)

**Table 3:**
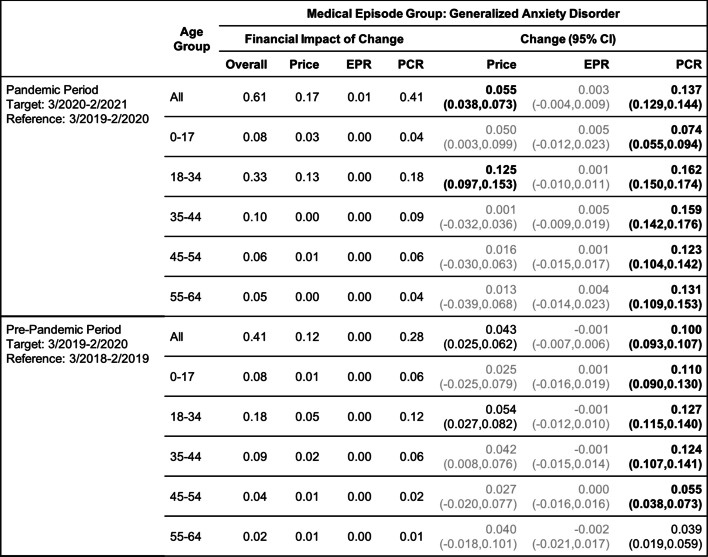
Changes of Mental Health Episodes in Twelve-Month Periods: Generalized Anxiety Disorder

Change in Price, EPR, and PCR: boldface, strong statistical significance (absolute value of Z score greater than 5, or *p*-value < 5.7e-7); lightface, weak or no statistical significance (absolute value of Z score less than or equal to 3, or *p*-value > 0.0027)

**Table 4:**
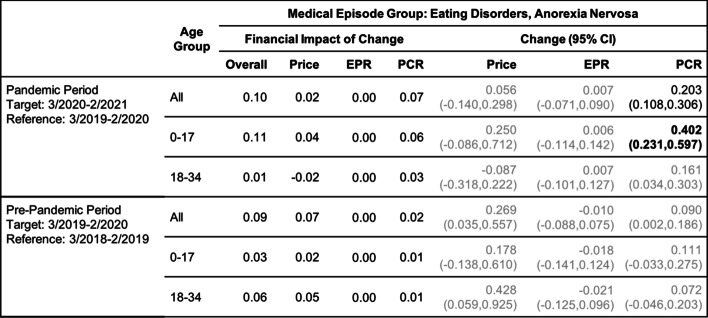
Changes of Mental Health Episodes in Twelve-Month Periods: Eating Disorders, Anorexia Nervosa

Change in Price, EPR, and PCR: boldface, strong statistical significance (absolute value of Z score greater than 5, or *p*-value < 5.7e-7); lightface, weak or no statistical significance (absolute value of Z score less than or equal to 3, or *p*-value > 0.0027)

**Table 5:**
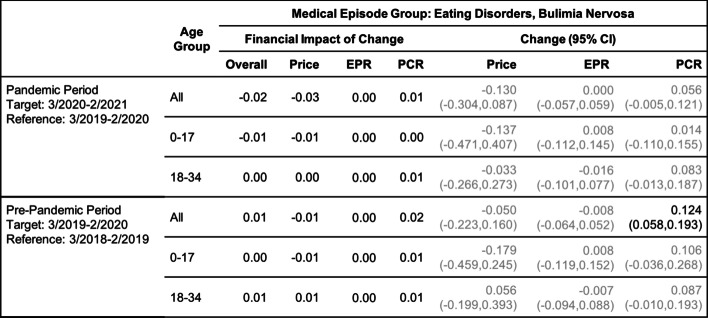
Changes of Mental Health Episodes in Twelve-Month Periods: Eating Disorders, Bulimia Nervosa

Change in Price, EPR, and PCR: boldface, strong statistical significance (absolute value of Z score greater than 5, or *p*-value < 5.7e-7); lightface, weak or no statistical significance (absolute value of Z score less than or equal to 3, or *p*-value > 0.0027)

**Table 6:**
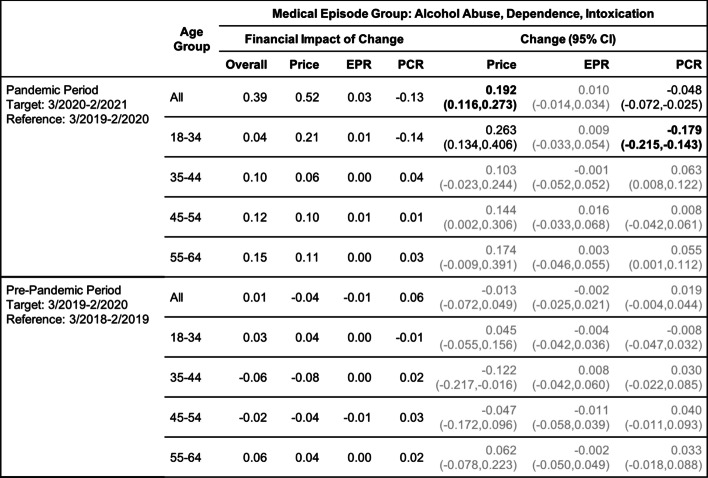
Changes of Mental Health Episodes in Twelve-Month Periods: Alcohol Abuse, Dependence, Intoxication

Change in Price, EPR, and PCR: boldface, strong statistical significance (absolute value of Z score greater than 5, or *p*-value < 5.7e-7); lightface, weak or no statistical significance (absolute value of Z score less than or equal to 3, or *p*-value > 0.0027)

**Table 7:**
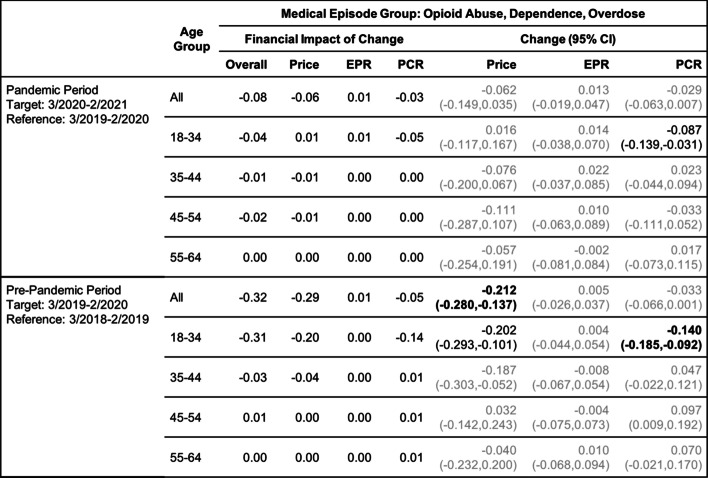
Changes of Mental Health Episodes in Twelve-Month Periods: Opioid Abuse, Dependence, Overdose

Change in Price, EPR, and PCR: boldface, strong statistical significance (absolute value of Z score greater than 5, or *p*-value < 5.7e-7); lightface, weak or no statistical significance (absolute value of Z score less than or equal to 3, or *p*-value > 0.0027)

Tables 8, 9, 10, 11, 12 and 13 contain the results of the quarterly analysis, also for patients in all age groups combined, where changes in seven 3-month periods during the pandemic relative to their references are compared with changes in the respective pre-pandemic periods relative to their references.

All changes discussed in the following are deemed statistically significant according to the results in Tables 2, 3, 4, 5, 6, 7, 8, 9, 10, 11, 12 and 13.

**Table 8:**
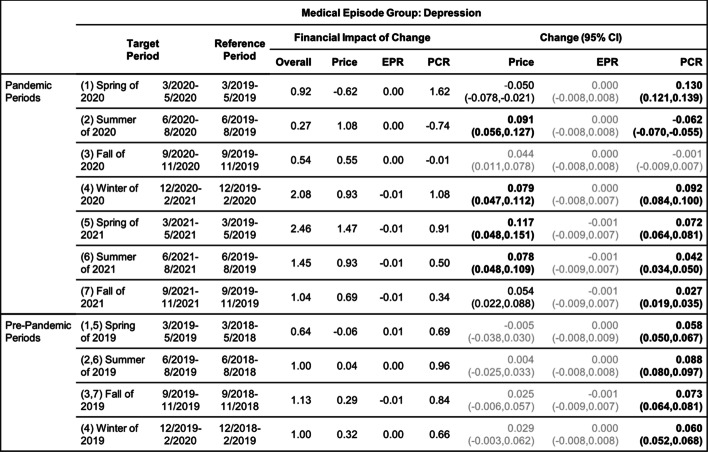
Changes of Mental Health Episodes in Three-Month Periods: Depression

Change in Price, EPR, and PCR: boldface, strong statistical significance (absolute value of Z score greater than 5, or *p*-value < 5.7e-7); lightface, weak or no statistical significance (absolute value of Z score less than or equal to 3, or *p*-value > 0.0027)

**Table 9:**
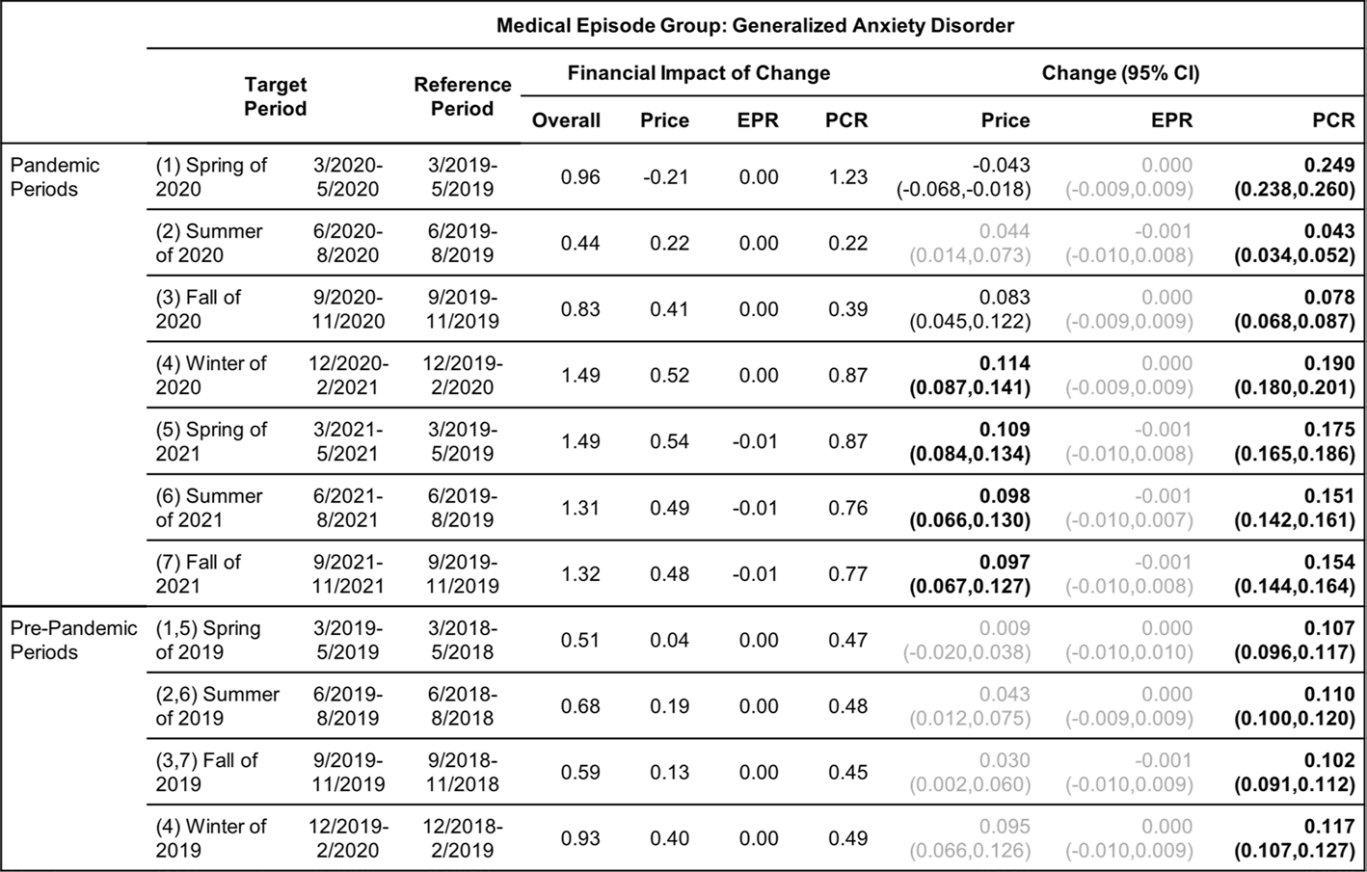
Changes of Mental Health Episodes in Three-Month Periods: Generalized Anxiety Disorder

Change in Price, EPR, and PCR: boldface, strong statistical significance (absolute value of Z score greater than 5, or *p*-value < 5.7e-7); lightface, weak or no statistical significance (absolute value of Z score less than or equal to 3, or *p*-value > 0.0027)

**Table 10:**
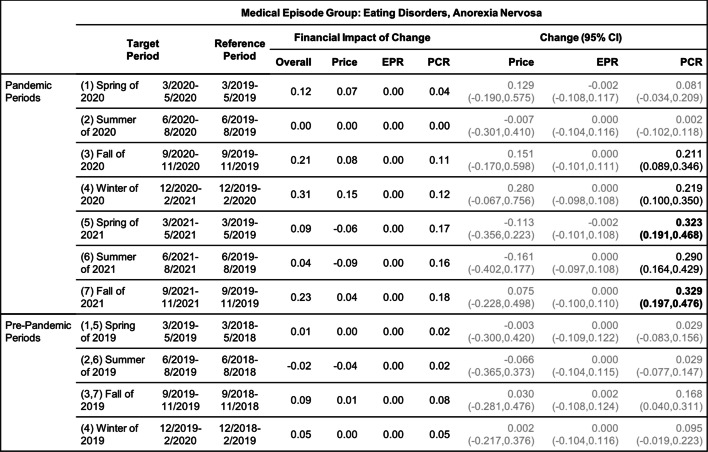
Changes of Mental Health Episodes in Three-Month Periods: Eating Disorders, Anorexia Nervosa

Change in Price, EPR, and PCR: boldface, strong statistical significance (absolute value of Z score greater than 5, or *p*-value < 5.7e-7); lightface, weak or no statistical significance (absolute value of Z score less than or equal to 3, or *p*-value > 0.0027)

**Table 11:**
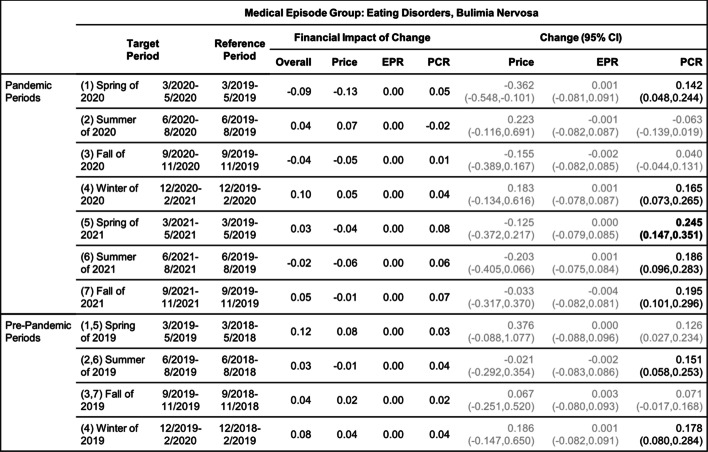
Changes of Mental Health Episodes in Three-Month Periods: Eating Disorders, Bulimia Nervosa

Change in Price, EPR, and PCR: boldface, strong statistical significance (absolute value of Z score greater than 5, or *p*-value < 5.7e-7); lightface, weak or no statistical significance (absolute value of Z score less than or equal to 3, or *p*-value > 0.0027)

**Table 12:**
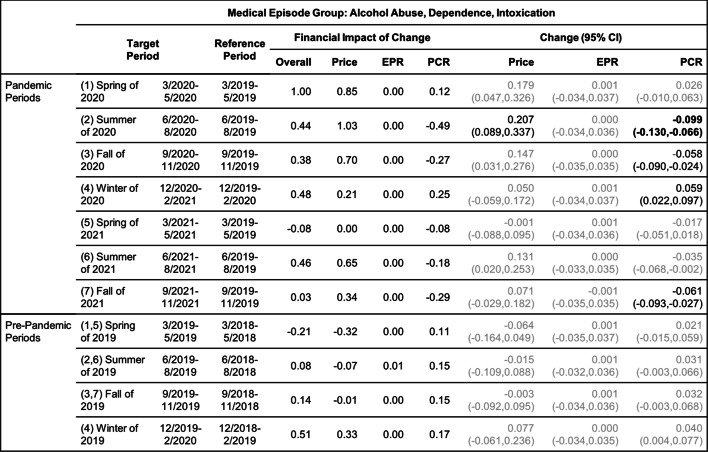
Changes of Mental Health Episodes in Three-Month Periods: Alcohol Abuse, Dependence, Intoxication

Change in Price, EPR, and PCR: boldface, strong statistical significance (absolute value of Z score greater than 5, or *p*-value < 5.7e-7); lightface, weak or no statistical significance (absolute value of Z score less than or equal to 3, or *p*-value > 0.0027)

**Table 13:**
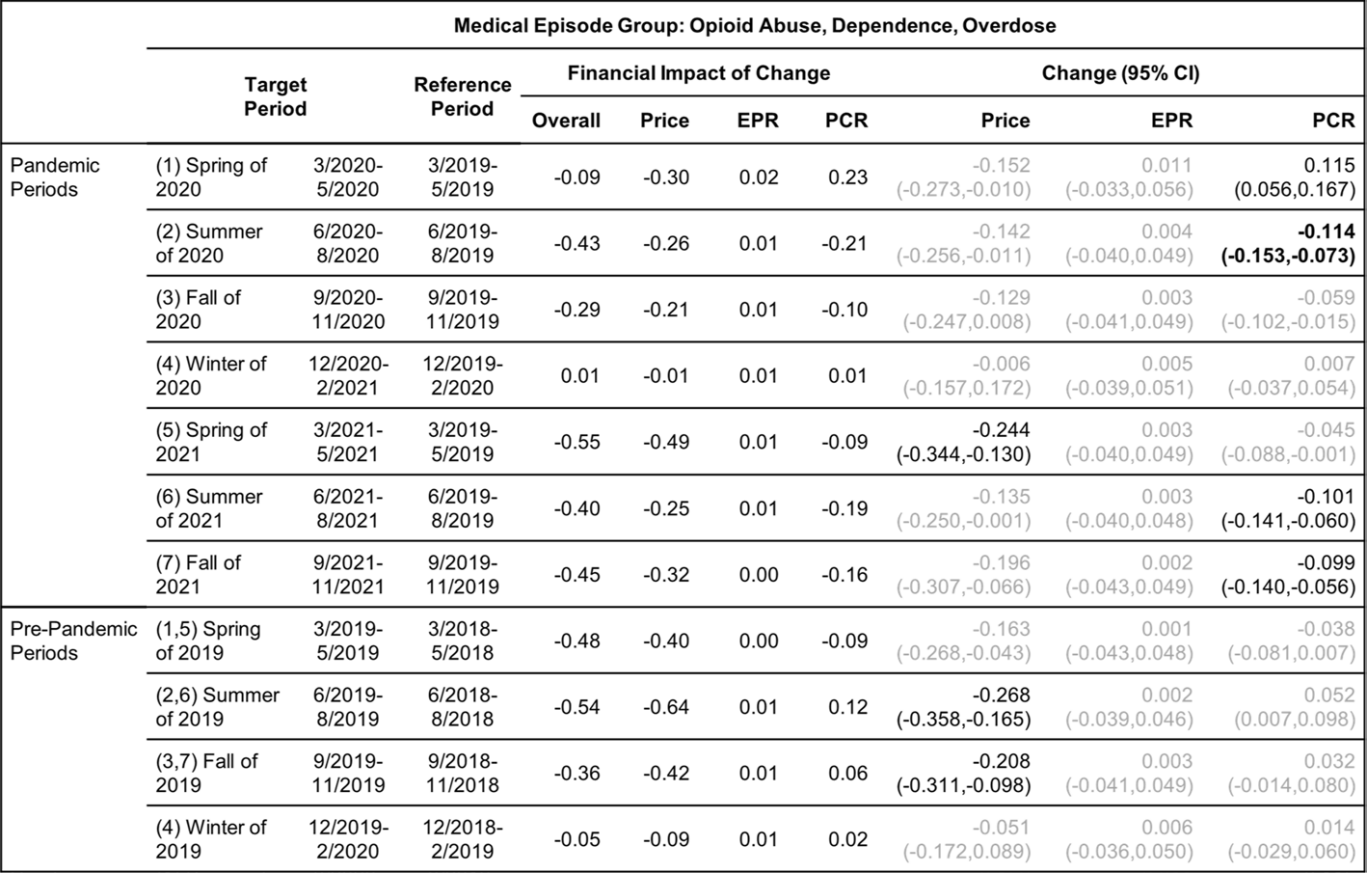
Changes of Mental Health Episodes in Three-Month Periods: Opioid Abuse, Dependence, Overdose

Change in Price, EPR, and PCR boldface, strong statistical significance (absolute value of Z score greater than 5, or *p*-value < 5.7e-7); lightface, weak or no statistical significance (absolute value of Z score less than or equal to 3, or *p*-value > 0.0027)

### Depression and generalized anxiety disorder

#### Yearly analysis

**Fig. 2:**
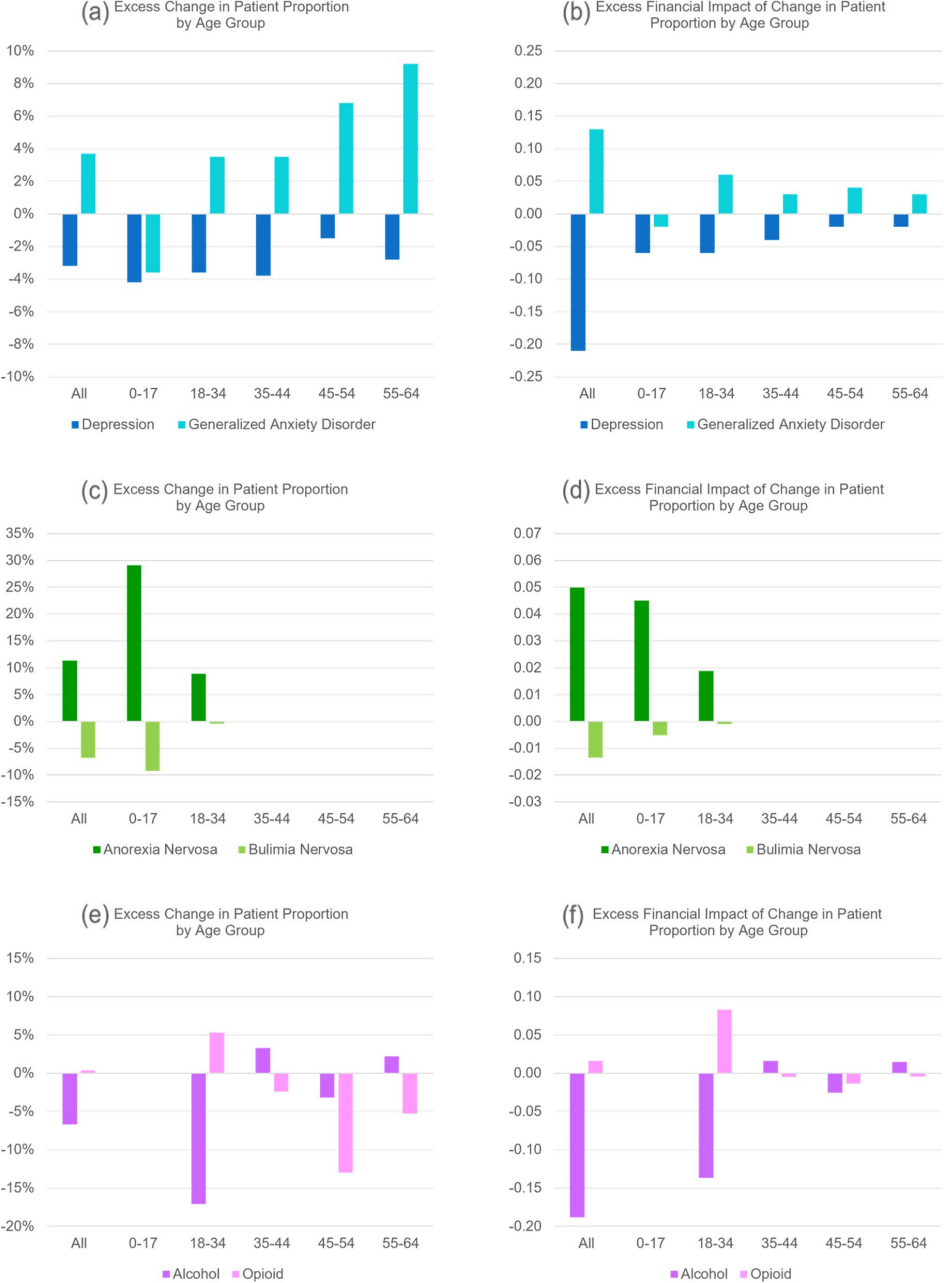
(**a**) and (**b**): excess change and excess financial impact of change in patient proportion (PCR) of depression and generalized anxiety disorder episodes by age group in the pandemic period 3/2020–2/2021 when compared to the baseline in the pre-pandemic period 3/2019–2/2020. (**c)** and (**d**): same for anorexia nervosa and bulimia nervosa episodes. (**e**) and (**f**): same for alcohol and opioid abuse episodes

In the yearly analysis (Tables 2 and 3), increases in patient proportion of depression and anxiety for all age groups combined are detected in both pandemic and pre-pandemic periods. The patient proportion of anxiety grew 13.7% in the pandemic period versus 10.0% in the pre-pandemic period. This, along with a higher growth in price per episode (5.5% versus 4.3%), resulted in a greater increase in per claimant expenditure ($0.61 versus $0.41 per month). The patient proportion of depression grew 3.7% in the pandemic period versus 6.9% in the pre-pandemic period, but per claimant expenditure grew by the same amount due to a 4.8% increase in price per episode. As a result of these changes in patient proportion, the excess change and excess financial impact of change (Figs. 2a and b) are negative for depression but positive for anxiety.

When broken down into age groups, depression for all age groups had a smaller increase in patient proportion in the pandemic period; anxiety had a greater increase in patient proportion for all age groups except age group 0–17 where the increase is smaller.

#### Quarterly analysis

In the quarterly analysis of patients in all age groups combined (Tables 8 and 9), there was an increase in all seven pandemic periods in patient proportion of anxiety; these increases played a major role in driving up the overall per claimant cost in all periods. A decrease in patient proportion of depression was detected only in the summer of 2020 (period 2), and the remaining periods had either an increase (periods 1 and 4–7) or no change (period 3).

**Fig. 3:**
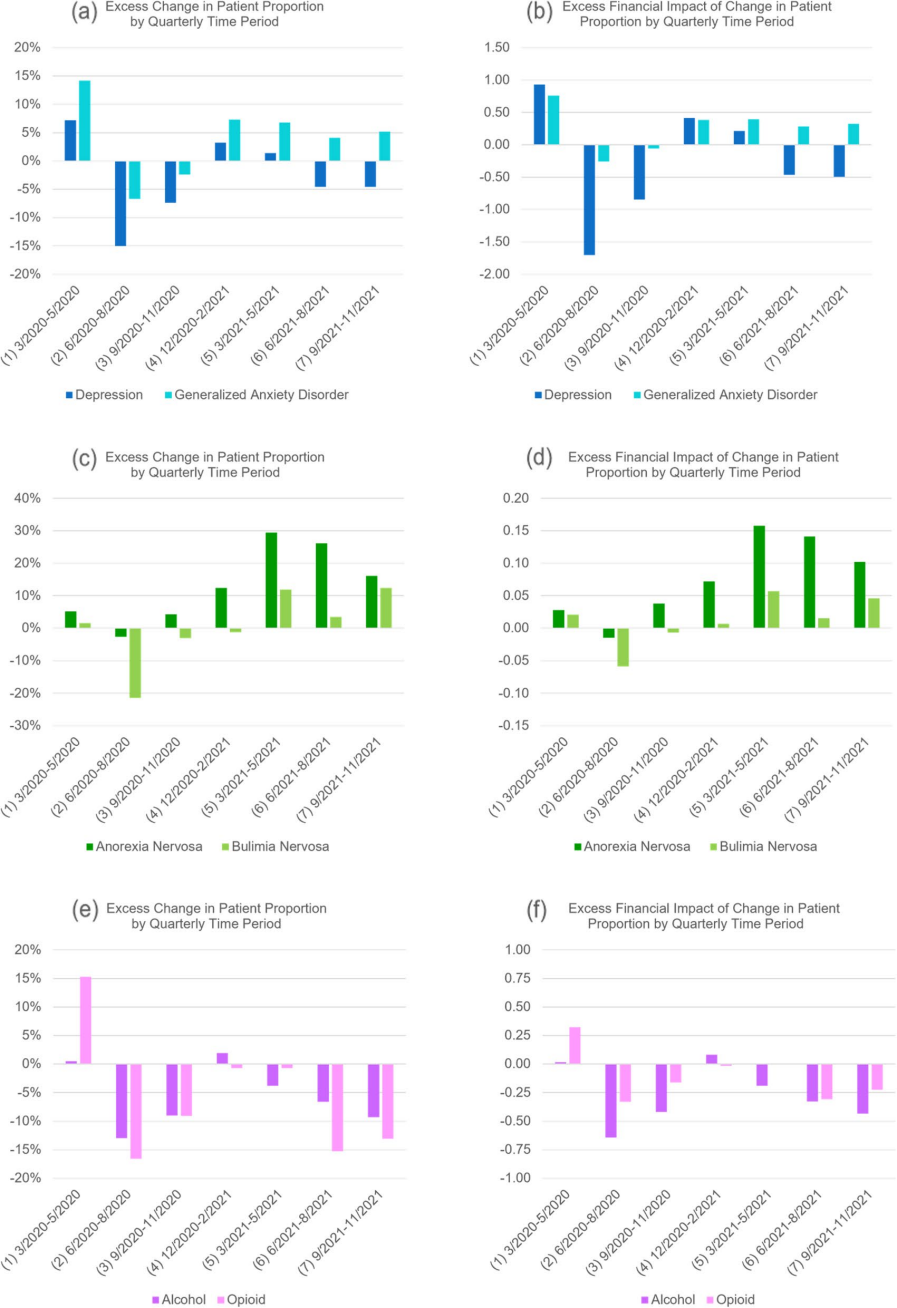
(**a**) and (**b**): excess change and excess financial impact of change in patient proportion (PCR) of depression and generalized anxiety disorder episodes in six periods during the pandemic when compared to the baseline in the respective periods before the pandemic. (**c**) and (**d**): same for anorexia nervosa and bulimia nervosa episodes. (**e**) and (**f**): same for alcohol and opioid abuse episodes

For anxiety, the observed excess change and excess financial impact of change in patient proportion (Figs. 3a and b) were positive in all periods except the summer and fall of 2020 (periods 2 and 3); the nearly 15% excess change in the spring of 2020 (period 1) was a main contributor to the overall positive excess change for the following 12-month pandemic period (Fig. 2a). In contrast, the observed excess change and excess financial impact of change in patient proportion of depression (Figs. 3a and b) were negative in the summer and fall of 2020 (periods 2 and 3) and in the summer and fall of 2021 (periods 6 and 7); the nearly -15% excess change in the summer of 2020 (period 2) was the main contributor to the overall negative excess change for the 12-month pandemic period (Fig. 2a).

The price per episode of depression and anxiety increased in all quarterly pandemic periods except the spring of 2020 (period 1). The increased price per episode of depression in the summer of 2020 (period 2) drove the overall expenditure up despite a decrease in patient proportion. The price per episode of anxiety played a lesser role in the overall financial impact.

### Eating disorders

#### Yearly analysis

In the yearly analysis for anorexia (Table 4), a 20.3% increase in patient proportion was detected in the pandemic period for patients in all age groups combined; the increase jumped to 40.2% for patients under age 18. The observed excess change increased from 10% for all age groups combined to 30% for patient under age 18 (Fig. 2c); the observed excess financial impact did not exhibit such differences (Fig. 2d).

For bulimia (Table 5), an increase in patient proportion was detected in the pre-pandemic period but not in the pandemic period.

#### Quarterly analysis

The quarterly analysis (Table 10) detected a patient proportion increase of anorexia in the fall of 2020 and thereafter (periods 3–7) but not in the earlier periods of the pandemic; the observed excess change and excess financial impact of change (Figs. 3c and d) became much higher in the winter of 2020 and thereafter (period 4–7).

The quarterly analysis (Table 11) also detected a patient proportion increase of bulimia in the spring of 2020 (period 1) in addition to the winter of 2020 and thereafter (periods 4–7); the over -20% excess change in the summer of 2020 (Fig. 3c, period 2) determined the overall negative excess change for the 12-month pandemic period (Fig. 2c).

### Alcohol and opioid use

#### Yearly analysis

For the yearly analysis (Table 6), a 4.8% decrease in the patient proportion for treating alcohol use was detected in the pandemic period when all age groups were combined; this decrease was accompanied by a 19.2% increase in price per episode, resulting in a greater increase in the overall expenditure compared to the pre-pandemic period ($0.36 versus $0.01). For patients in age group 18–34, there was a more pronounced 17.9% decrease in patient proportion, together with a 26.3% increase in price per episode; the observed excess change for patient proportion was over -15% (Fig. 2e).

For the same age group, a decrease in patient proportion for treating opioid use was also detected in the pandemic period (Table 7), but the observed excess change and excess financial impact of change were positive (Figs. 2e and f).

#### Quarterly analysis

For the quarterly analysis (Table 12), a decrease in patient proportion for treating alcohol use was detected in the summer and fall of 2020 (periods 2 and 3) which largely determined the overall negative excess change of patient proportion in the 12-month pandemic period (Fig. 2e). In the summer of 2020 (period 2), a 20.7% increase in price per episode dominated a 9.9% decrease in patient proportion and drove up the per claimant expenditure in this period by $0.44.

For treating opioid use (Table 13), an increase in patient proportion was detected earlier in the spring of 2020 (period 1); the observed excess change in this period was over 15% (Fig. 3e), but it was offset by a decrease in the summer of 2020 (period 2), resulting in a small positive excess change in the yearly assessment in the pandemic period (Fig. 2e). In the remaining quarterly periods, the observed excess change and excess financial impact of change in patient proportion (Figs. 3e and f) were all negative for treating opioid use.

## Discussion

Increases in the prevalence of mental health conditions during the COVID-19 pandemic have been widely reported in the literature. This study demonstrates that the COVID-19 pandemic impacted the use patterns and expenditures for patients receiving mental health services. These patterns are complex and remain a subject for intensive research. Our findings contribute to this effort. Specifically, this study measures the impact of three fundamental drivers of the expenditure changes in mental health episodes: price, use, and intensity of services. Overall, when there were changes in the cost of mental health episodes, these were largely driven by changes in the proportion of total claimants using these services out of all healthcare claimants (patient proportions). For some mental health conditions, we observed decreases in costs because of decreases in the proportion of patients with that condition (e.g., patients with depression and patients being treated for alcohol). In a few cases, the price of the episode also contributed to the cost change (e.g., treatment for alcohol). This study also assessed how cost changes changed in pre-pandemic time periods compared to post-pandemic onset time periods. The study tries to understand whether the COVID-19 pandemic contributed to the changes in trend observed. Much like the financial impacts, changes in patient proportions of a condition also contributed to the changes in trend. Lastly, this study analyzed quarterly segments of time following the onset of the pandemic to understand if patterns of use of healthcare services and expenditures were impacted by the time elapsed from the pandemic onset in March 2020.

Significant prevalence increases of anxiety and depression in the general population have been reported in the literature [4, 5, 29, 30]. Our analysis of MarketScan® data shows that the proportion of patients receiving anxiety treatment (except for those under 18 years of age) among all claimants experienced a greater increase in the first year of the pandemic (3/2020–2/2021) than in the year before it started. However, for this same pandemic period, the proportion of patients receiving depression treatment had a smaller increase than in the year before the pandemic. Further studies are needed to explain why these proportions behaved differently during the pandemic, especially whether telemedicine played different roles for these patients [31].

The smaller increase in the proportion of patients aged 18 and younger for anxiety treatment may indicate an unmet need in this age group. Future research should understand the drivers of this unmet need (e.g., access issues with schools being closed, stigma issues, etc.) especially given the increased use of services for anorexia in this age group. The greater increase in the proportion of patients for anxiety treatment in the summer and fall of 2021 (periods 6 and 7), in contrast with a smaller increase in the summer of 2020 (period 2), may be indicative of a more prolonged need for anxiety services.

Prices per episode for depression and anxiety grew more than expected during the pandemic. Since the analysis suggests the use of services for depression and anxiety may remain high, health insurers may want to focus on ways to control price as a mechanism to address rising costs for depression and anxiety episodes.

Prevalence increases of eating disorders during the pandemic have been reported in the literature [14, 32, 33]. Our analysis shows that patients receiving treatment for anorexia accounted for a higher-than-expected proportion of all claimants only after the fall of 2020 (period 3) rather than immediately after the outbreak of the pandemic (period 1). Patients receiving treatment for bulimia had a similarly delayed increase. Future research is needed to investigate reasons for this phenomenon, for example, whether it was caused by limited accessibility for traditional treatment of eating disorders in the beginning of the pandemic [9, 34, 35].

According to a Rand study [18], adult consumption of alcohol increased 14% during the pandemic. Our study shows that patients receiving treatment for alcohol use, largely driven by patients in age group 18–34, accounted for a lower-than-expected proportion among all claimants after the outbreak of the pandemic, and the proportion has stayed low since after initial lockdowns. Additional studies are needed to explain this decrease against previously reported increase in alcohol consumption [16, 36], although it could also be explained by unmet needs [18, 37].

The COVID-19 pandemic proved to be a unique challenge for treating opioid dependence conditions. In the US, the Substance Abuse and Mental Health Services Administration (SAMHSA) went so far as to change treatment guidelines to address possible disruptions in care [19]. Our study shows that in the first three months after the outbreak of the pandemic, the proportion of patients receiving treatment for opioid use among all claimants was higher than expected compared to the same period in the previous year. This may be attributed in part to the ability of these patients to leverage telehealth services [38–40] especially in comparison with patients in need of treatment for alcohol use. However, similar to alcohol use, the time periods following that initial lockdown period show a decrease or no change in the proportion of patients receiving treatment for opioid use relative to the pre-pandemic periods. Further research is needed to understand the implication of these patterns along with similar findings [41], but such unmet needs might explain the overall reported increase in opioid abuse related deaths [42].

Overall, we think that the unmet needs here described could be a consequence of the healthcare system not being ready to support a society in lockdown and that even an initial increase in telehealth usage [20, 39] could not replace in-person treatment.

### Limitations

MarketScan® Database only represents a commercially insured population, so any generalization to a different population should be considered with care. Medical episodes built by the MEG methodology are based on primary diagnosis and may undercount claims where mental health conditions were not primary diagnoses or missing from the diagnosis list. A depression episode may be absorbed into a major depressive episode of bipolar disorder if they occurred in close proximity of time, and the latter group was excluded from this analysis. In this paper, we limited the scope of analysis to three fundamental factors that contribute to the changes in cost and use patterns. Additional variables such as the place of service, the treatment, the severity of the condition may also play a role in these changes and deserve to be reported in further research. Finally, our analysis excluded claims with missing financial variables.

## Conclusions

Based on a cross-sectional analysis of health insurance claims in MarketScan® Commercial Database, this study identified changes in use patterns and expenditures for six common mental health conditions. These changes and their financial impacts vary across conditions and periods of the pandemic. Some use patterns were unexpected from previously reported prevalence increases among the general population, including a smaller increase for treatment of depression, a decrease for treatment of alcohol use, no significant change in treatment of opioid use, and a delayed increase for treatment of anorexia. This observational analysis provides insights for health insurers, service providers, and researchers on the impact of the COVID-19 pandemic on mental health services in a commercially-insured population and points to several potential unmet treatment needs.

## Supplementary Information

**Additional file 1:** **Supplementary Table 1.** ICD Diagnosis Codes Used to Construct Mental Health Episodes. **Supplementary Figure 1.** (a) and (b): observed change in the pandemic period 3/2020-2/2021 and the pre-pandemic period 3/2019-2/2020 for patients in all age groups combined. (c) and (d): observed financial impact of change in these periods. **Supplementary Table 2.** Trending of Changes Detected in Twelve-Month Periods Before and After the Pandemic Outbreak for Patients in All Age Groups Combined.

## Data Availability

MarketScan® data are commercially available (https://www.merative.com/content/dam/merative/documents/brief/Marketscan_explainer_general.pdf.coredownload.inline.pdf).
