# Supplementary material for: Impact of the COVID-19 pandemic on treatment for mental health needs: a perspective on service use patterns and expenditures from commercial medical claims data
Source: BMC Health Serv Res. 2023 Feb 16;23:163. doi: 10.1186/s12913-023-09080-9 (PMC9932413; doi:10.1186/s12913-023-09080-9)

**Impact of the COVID-19 Pandemic on Treatment for Mental Health Needs: A Perspective on Service Use Patterns and Expenditures from Commercial Medical Claims Data – Supplementary Material**

Ta-Hsin Li^1*^, PhD; Leah Kamin^2^, MPH; Judy George^2^, PhD; Fernando Suarez Saiz^2^, MD; Pablo Meyer^1^, PhD

^1^ IBM Thomas J. Watson Research Center, Yorktown Heights, NY 10598

^2^ IBM Watson Health, Cambridge, MA 02142

*Corresponding author: IBM Thomas J. Watson Research Center, Yorktown Heights, NY 10598-0218 ([thl@us.ibm.com](mailto:thl@us.ibm.com))

1. ICD Diagnosis Codes Considered in Mental Health Episodes

Medical episodes describe a patient’s complete course of care for a single illness or condition, considering not only claims with disease-specific diagnosis codes but also those with nonspecific diagnosis codes or those with no diagnosis codes (e.g., pharmacy claims) yet deemed relevant to a mental health episode. In the following table contains the ICD-CM-9 and ICD-CM-10 diagnosis codes used by the Medical Episode Grouper (MEG) to build mental health episode groups discussed in the main article.

Supplementary Table 1. ICD Diagnosis Codes Used to Construct Mental Health Episodes


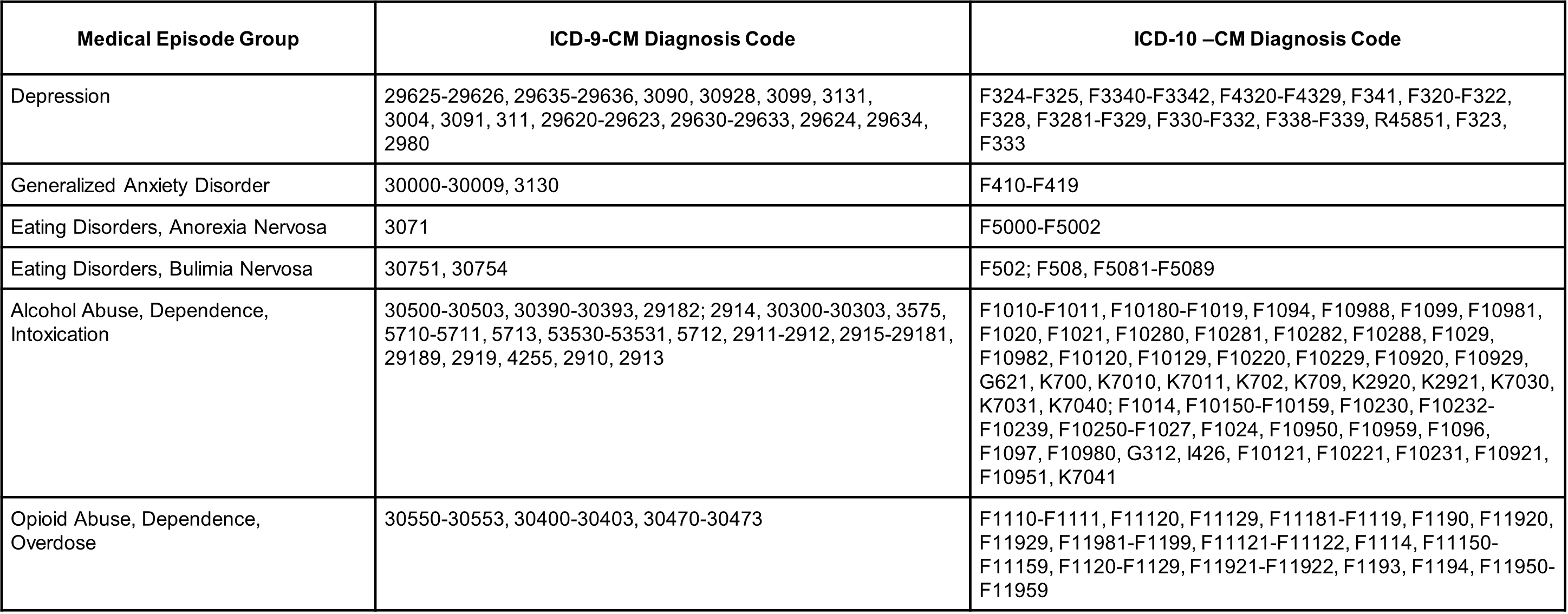


2. Change and Financial Impact Calculation Details.

The three factors, Price, EPR, and PCRE, contribute to Cost in the following way:

Cost = Price × EPR × PCR.

For a given contributing factor (Price, EPR, or PCR) to Cost, we calculate its value in a target period and its value in a reference period. The difference between these quantities as a fraction (or percentage) of the reference value is called the change in this contributing factor. For example, the change in patient proportion in target period March-May 2020 relative to the reference period March-May 2019 is given by

[PCR(March-May 2020) - PCR(March-May 2019)] ÷ PCR(March-May 2019).

The financial impact of this change is defined as the increment of Cost when this factor takes on the value in the target period while the remaining two factors are held at their values in the reference period. For example, the financial impact due to the change in patient proportion in March-May 2020 relative to March-May 2019 is defined by

Price(March-May 2019) × EPR(March-May 2019) × PCR(March-May 2020)

- Cost(March-May 2019).

3. Statistical Hypothesis Testing for Change Detection

To detect changes in these factors, we employ a *Z-*test procedure under the null hypothesis of no change, where the *Z* score of a factor is defined as the observed log ratio of this factor in the target period relative to the reference period, normalized by its standard deviation based on the mathematical properties of log ratios of Gaussian means (for Price), Poisson or negative binomial rates (for EPR), and binomial proportions (for PCR) from two random samples [25-28]. Depending on the detection threshold of this procedure, the statistical significance of a change will be presented in three categories: strong (*Z* score is greater than 5 in absolute value, equivalent to *p*-value < 5.7e-7), moderate (*Z* score is greater than 3 but less than or equal to 5 in absolute value, equivalent to 5.7e-7 ≤ *p*-value < 0.0027), or weak or no change (*Z* score is less than or equal to 3 in absolute value, equivalent to *p*-value > 0.0027).

4. Yearly Analysis for Patients in All Age Groups Combined (Table 2 in the Main Text)


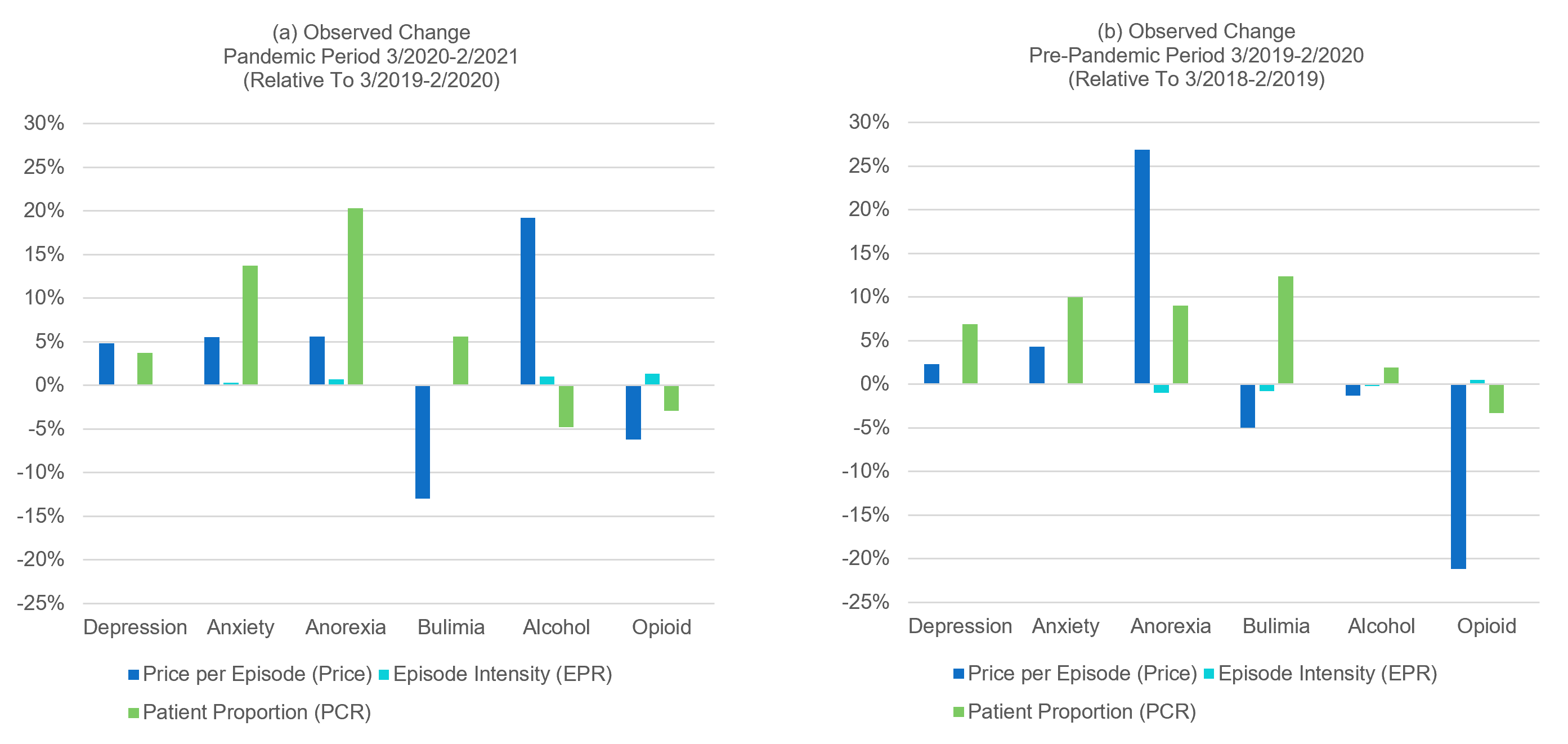


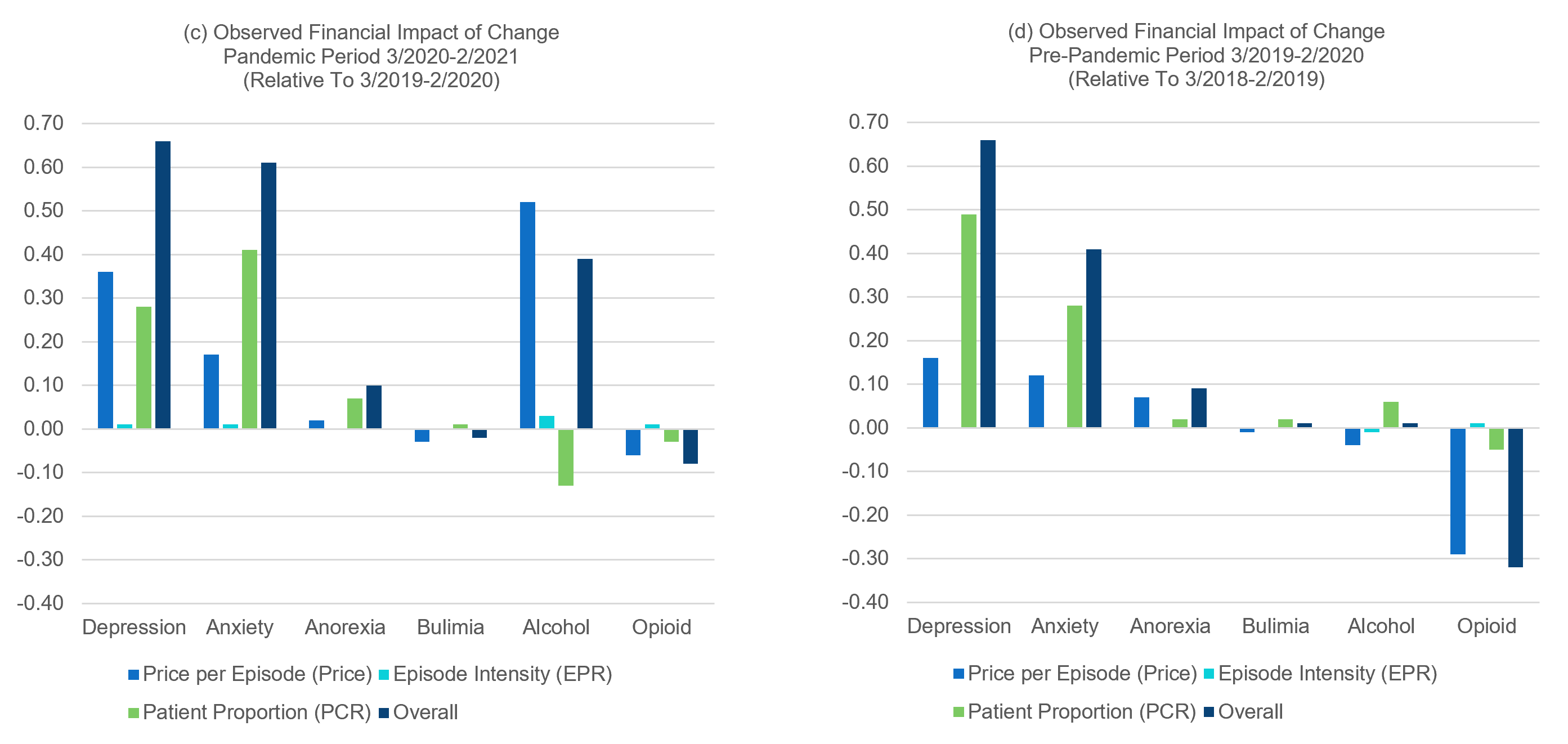


Supplementary Figure 1. (a) and (b): observed change in the pandemic period 3/2020-2/2021 and the pre-pandemic period 3/2019-2/2020 for patients in all age groups combined. (c) and (d): observed financial impact of change in these periods.

Supplementary Table 2. Trending of Changes Detected in Twelve-Month Periods Before and After the Pandemic Outbreak for Patients in All Age Groups Combined


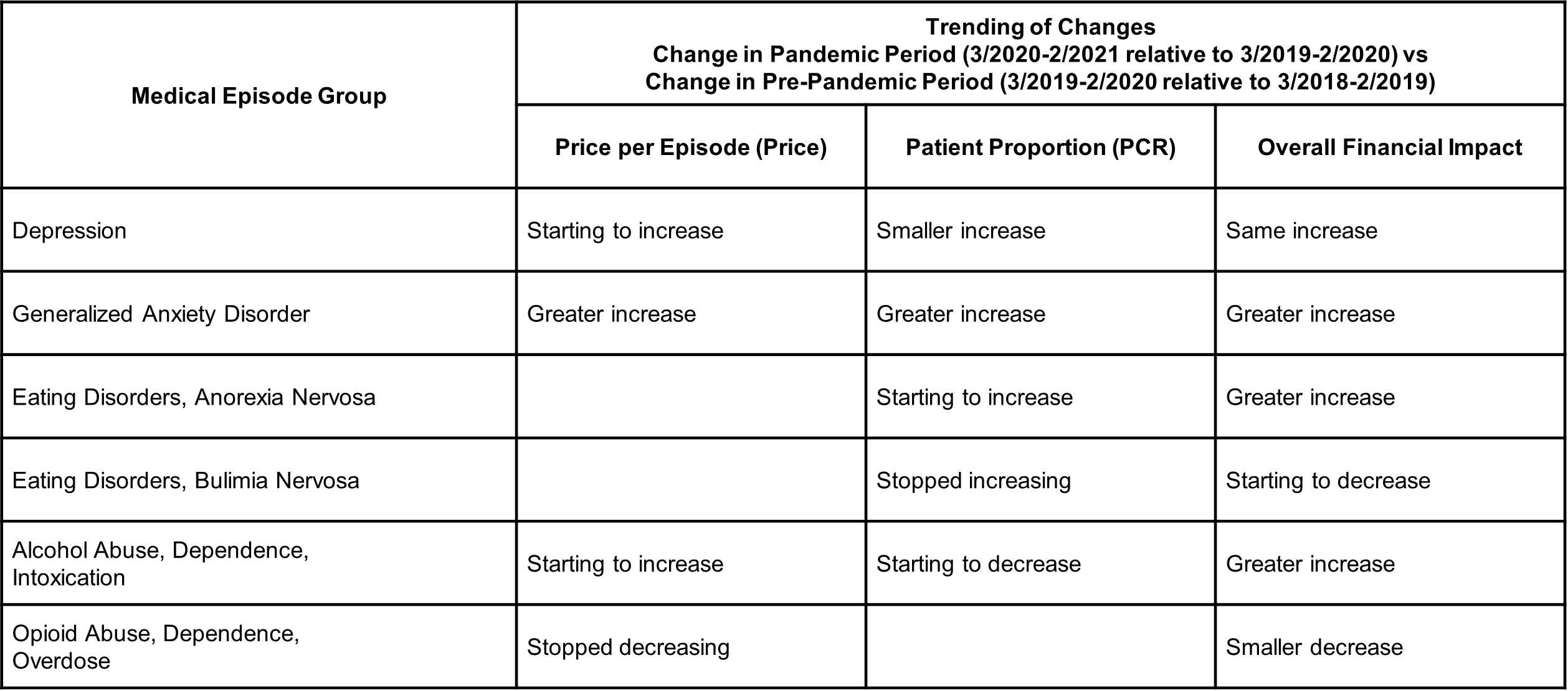

Supplement: Supplementary file 1 — Additional file 1: Supplementary Table 1. ICD Diagnosis Codes Used to Construct Mental Health Episodes. Supplementary Figure 1. (a) and (b): observed change in the pandemic period 3/2020-2/2021 and the pre-pandemic period 3/2019-2/2020 for patients in all age groups combined. (c) and (d): observed financial impact of change in these periods. Supplementary Table 2. Trending of Changes Detected in Twelve-Month Periods Before and After the Pandemic Outbreak for Patients in All Age Groups Combined. [file 12913_2023_9080_MOESM1_ESM.docx]
